# Genome Sequencing of Foot-and-Mouth Disease Virus Type O Isolate GRE/23/94

**DOI:** 10.1128/genomeA.00353-16

**Published:** 2016-05-12

**Authors:** David J. King, Nick J. Knowles, Graham L. Freimanis, Donald P. King

**Affiliations:** The Pirbright Institute, Pirbright, Woking, Surrey, United Kingdom

## Abstract

The complete genome of a foot-and-mouth disease type O virus originating from an epidemic in Greece in 1994 is reported. This virus belongs to the Middle East-South Asia topotype.

## GENOME ANNOUNCEMENT

Foot-and-mouth disease (FMD) virus belongs to the genus *Aphthovirus* within the family *Picornaviridae* and causes FMD in cloven-hooved animals. FMD virus has a positive-sense single-stranded RNA genome of approximately 8.3 kb, which contains a single open reading frame (ORF) encoding 4 structural and 10 nonstructural proteins (1).

The 1994 FMD virus (FMDV) epidemic in Greece began with high morbidity in sheep. After approximately 1 month there was a sharp decline of clinical cases within sheep flocks, while morbidity within cattle and pig populations remained stable. There is evidence to suggest that FDMV cannot maintain itself within sheep populations for long periods of time (2), and therefore the full genome of a representative field isolate (GRE/23/94) was determined to promote understanding of whether there were any genomic determinates of host specificity for these outbreaks.

O/GRE/23/94 was obtained from the World Reference Laboratory for FMD (WRLFMD) collection (The Pirbright Institute, United Kingdom) and was isolated, using primary calf thyroid cells, from ovine epithelium collected on 4 August 1994 in Xanthi during the peak of the Greek FMD epidemic (2). RNA was extracted using an RNeasy minikit (Qiagen) and Illumina sequencing was performed as previously described (3). Raw FASTQ files were filtered using Sickle (4), with reads below a quality score of Q30 and a length of 25 nucleotides removed. Consensus sequences were generated using the *de novo* assembler program IDBA UD (version 1.1.1) (5), with an optimum k-mer length determined within the program. FMDV sequences were identified from the contigs produced by a BLAST search and assembled using BioEdit (version 7.2.5). Using the generated consensus sequence as a reference, BWA-MEM (version 0.7.11) (6) was used for alignment while SAMtools (version 1.3) (7) was used to process SAM/BAM files. Alignments were visually checked using Tablet (8), coverage plots were generated using BEDTools (version 2.25) (9), and a final consensus sequence was generated by use of DiversiTools (https://github.com/josephhughes/btctools). The total length of the genome recovered was 8,161 nucleotides with a mean coverage depth of 1,634 across the genome; we were not able to determine the sequence or length of the poly(C) tract or 7 adjacent downstream nucleotides. Comparison of O/GRE/23/94 with nine sequences belonging to the Middle East-South Asia topotype (10) (AJ539136, AJ539138, AJ539141, AY593823, AY593828, DQ404171, HQ268524, JX040493, and KT003716) showed no indels within the polyprotein ORF; however, a deletion of 43 nucleotides within the 5′ untranslated region (located at position 410) was shared with AY593828 and KT003716, relative to the other seven sequences. We hypothesize that this represents the deletion of one of four pseudoknots of unknown function commonly present in other FMDV genomes. Additionally, a cluster of seven amino acid substitutions, different from the nine other sequences, was found in the carboxy-terminal half of the 3A polypeptide, which has been implicated in host specificity (11–13). The VP1 sequence of O/GRE/23/94 was identical to that previously reported (14).

### Nucleotide sequence accession number.

The nucleotide sequence for FMDV O/GRE/23/94 has been deposited in GenBank under accession number KU726614.

## References

[1] Grubman MJ, Baxt B 2004 Foot-and-mouth disease. Clin Microbiol Rev 17:465–493. doi:10.1128/CMR.17.2.465-493.2004. 15084510 PMC387408

[2] Hughes GJ, Mioulet V, Haydon DT, Kitching RP, Donaldson AI, Woolhouse MEJ 2002 Serial passage of foot-and-mouth disease virus in sheep reveals declining levels of viraemia over time. J Gen Virol 83:1907–1914. doi:10.1099/0022-1317-83-8-1907. 12124454

[3] Logan G, Freimanis GL, King DJ, Valdazo-González B, Bachanek-Bankowska K, Sanderson ND, Knowles NJ, King DP, Cottam EM 2014 A universal protocol to generate consensus level genome sequences for foot-and-mouth disease virus and other positive-sense polyadenylated RNA viruses using the Illumina MiSeq. BMC Genomics 15:828. doi:10.1186/1471-2164-15-828. 25269623 PMC4247156

[4] Joshi NA, Fass JN 2011 Sickle: A sliding-window, adaptive, quality-based trimming tool for FastQ files (Version 1.33) [Software]. Available at https://github.com/najoshi/sickle.

[5] Peng Y, Leung HCM, Yiu SM, Chin FYL 2012 IDBA-UD: a de novo assembler for single-cell and metagenomic sequencing data with highly uneven depth. Bioinformatics 28:1420–1428. doi:10.1093/bioinformatics/bts174. 22495754

[6] Li H, Durbin R 2010 Fast and accurate long-read alignment with Burrows-Wheeler transform. Bioinformatics 26:589–595. doi:10.1093/bioinformatics/btp698. 20080505 PMC2828108

[7] Li H, Handsaker B, Wysoker A, Fennell T, Ruan J, Homer N, Marth G, Abecasis G, Durbin R, Subgroup 1000. Genome Project Data Processing 2009 The sequence alignment/map format and SAMtools. Bioinformatics 25:2078–2079. doi:10.1093/bioinformatics/btp352. 19505943 PMC2723002

[8] Milne I, Stephen G, Bayer M, Cock PJA, Pritchard L, Cardle L, Shaw PD, Marshall D 2013 Using tablet for visual exploration of second-generation sequencing data. Brief Bioinform 14:193–202. doi:10.1093/bib/bbs012. 22445902

[9] Quinlan AR 2014 BEDTools: the Swiss-Army tool for genome feature analysis. Curr Protoc Bioinform 8:47. 10.1002/0471250953.bi1112s47 PMC4213956 25199790

[10] Knowles NJ, Samuel AR 2003 Molecular epidemiology of foot-and-mouth disease virus. Virus Res 91:65–80. doi:10.1016/S0168-1702(02)00260-5. 12527438

[11] Knowles NJ, Davies PR, Henry T, O’Donnell V, Pacheco JM, Mason PW 2001 Emergence in Asia of foot-and-mouth disease viruses with altered host range: characterization of alterations in the 3A protein. J Virol 75:1551–1556. doi:10.1128/JVI.75.3.1551-1556.2001. 11152528 PMC114061

[12] Núñez JI, Baranowski E, Molina N 2001 A single amino acid substitution in nonstructural Protein 3A can mediate adaptation of foot-and-mouth disease virus to the guinea pig. J Virol 75:3977–3983. 11264387 10.1128/JVI.75.8.3977-3983.2001 PMC114889

[13] Gladue DP, O’Donnell V, Baker-Bransetter R, Pacheco JM, Holinka LG, Arzt J, Pauszek S, Fernandez-Sainz I, Fletcher P, Brocchi E, Lu Z, Rodriguez LL, Borca MV 2014 Interaction of foot-and-mouth disease virus nonstructural protein 3A with host protein DCTN3 is important for viral virulence in cattle. J Virol 88:2737–2747. doi:10.1128/JVI.03059-13. 24352458 PMC3958104

[14] Knowles NJ, Samuel AR, Davies PR, Midgley RJ, Valarcher JF 2005 Pandemic strain of foot-and-mouth disease virus serotype O. Emerg Infect Dis 11:1887–1893. 16485475 10.3201/eid1112.050908 PMC3367651

